# Draft genome sequences of *Limosilactobacillus fermentum* IJAL 01 335, isolated from a traditional cereal fermented dough

**DOI:** 10.1128/mra.00868-25

**Published:** 2025-10-31

**Authors:** Marcel Houngbédji, Geoffroy Romaric Bayili, Maria Diaz, Amy Atter, Arjan Narbad, S. Wilfrid Padonou

**Affiliations:** 1 Laboratoire de Sciences et Technologies des Aliments, Faculté des Sciences Agronomiques, Université d’Abomey-Calavi, Jéricho, Cotonou, Benin; 2 Laboratoire de Sciences et Technologie des Aliments, des Bioressources et de Nutrition Humaine, Université Nationale d’Agriculture, Sakété, Benin; 3 Département Technologie Alimentaire (DTA), Institut de Recherche en Sciences Appliquées et Technologies (IRSAT), Centre National de la Recherche Scientifique et Technologique (CNRST) 206843, Ouagadougou, Burkina Faso; 4 Microbes and Food Safety Institute Strategic Programme, Quadram Institute Bioscience 7308, Norwich, United Kingdom; 5 Food Microbiology and Mushroom Research Division, CSIR-Food Research Institute 541874 , Accra, Ghana; 6 Food, Microbiome and Health Institute Strategic Programme, Quadram Institute Bioscience, Norwich Research Park 7308, Norwich, United Kingdom; State Key Laboratory of Food Science and Resources, Nanchang, Jiangxi, China

**Keywords:** lactic acid bacteria, cereal dough, spontaneous fermentation, draft genome sequence

## Abstract

*Limosilactobacillus fermentum* IJAL 01 335 was isolated from *mawè*, a spontaneously fermented cereal dough from Benin. The 1.83 Mb draft genome sequence (52.37% GC) comprises 154 contigs, 1,836 coding sequences, and 23 predicted antibiotic resistance genes, providing insights into its genetic features and potential application in food fermentation.

## ANNOUNCEMENT

*Limosilactobacillus fermentum* is a heterofermentative lactic acid bacterium found in fermented foods and the human gastrointestinal tract (1). Its widespread occurrence in traditional products, including sourdough (2), kefir (3), fermented vegetables (4), and dairy (5), reflects its ecological versatility and technological relevance in the production of plant- and milk-based products. In the human gut, *L. fermentum* has been associated with probiotic properties (6). *L. fermentum* was frequently reported as the predominant bacteria involved in the spontaneous fermentation of traditional cereal-based African products, including *mawè* (7), *koko* (8), or *ogi* (9). Therefore, whole-genome data from *L. fermentum* strains involved in such indigenous foods will contribute to the understanding of their metabolic capabilities and functional diversity.

*L. fermentum* IJAL 01 335 was isolated in Atlantique, Benin (2.0 latitude and 6.0 longitude) in July 2017, from *mawè*, a spontaneously fermented dough from maize in Benin (7). The dough was plated anaerobically with Anaerocult A (Merck KGaA, Darmstadt, Germany) on MRS agar plates (pH 6.8 ± 2, 48 h, 30°C), single colonies sub-cultured, and purified cultures stored at −80°C in 30% glycerol. For DNA extraction, the strain was revived by streaking on MRS agar plates, and a distinct colony was picked. Genomic DNA was extracted using a cetyltrimethylammonium bromide (CTAB)-based protocol, with RNase and proteinase K lysis followed by incubation at 65°C (10). Library preparation was performed using the Nextera XT DNA Library Prep Kit (Illumina, San Diego, CA, USA) according to the manufacturer’s instructions, without modifications, using 1 ng of input genomic DNA as recommended by the kit protocol. Sequencing (150 cycles) was conducted on the Illumina NextSeq platform (Earlham Institute, Norwich, UK) resulting in 3,870,856 raw reads. Reads were quality-trimmed (BBDuk v38.68), and those shorter than 100 bp or with an average quality score below Phred 20 were discarded. *De novo* assembly was performed (SPAdes v3.11.1) (11) and annotated using the National Center for Biotechnology Information-Prokaryotic Genome Annotation Pipeline (NCBI-PGAP v6.10, 2025) (12) through the NCBI Genome Submission Portal. The BV-BRC PATRIC web server (13) was used for complementary annotation using the RAST tool kit. Taxonomic classification was assigned as *L. fermentum* by the NCBI Prokaryotic Genome Annotation Pipeline (PGAP) and confirmed with the BV-BRC Similar Genome Finder Service (Mash/MinHash), with the closest reference strain corresponding to the genome in the BV-BRC database with the smallest Mash distance to our assembly (14). For visualization, the BV-BRC PATRIC circular genome viewer was used to display contigs in a circular layout without overlap identification/trimming and genome rotation, as the assembly remained in draft form.

**Fig 1:**
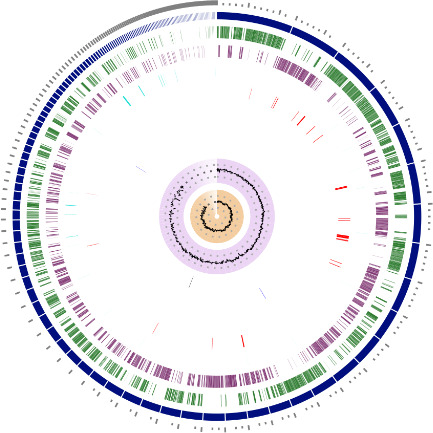
Circular representation of the draft genome of *Limosilactobacillus fermentum* IJAL 01 335. The genome remains in 154 contigs; no circularization was performed. Tracks are displayed as concentric rings, from outermost to innermost.

The assembled genome contains 154 contigs, coverage of 134×, total length 1,831,075 bp, average G + C content of 52.37% and N_50_ of 35,959 bp. The genomic features are represented in the circular map (Fig. 1). Based on the PGAP (v.6.10), this genome had 1,836 protein coding sequences, 51 transfer RNA genes, 3 ribosomal RNA genes, 23 antibiotic resistance genes, 4 ncRNAs, and 80 pseudogenes. The closest reference strain, with a distance of 0.01843 (Mash distance), was *L. fermentum* SCB0035 (GenBank accession: CP094655).

## Data Availability

This draft genome shotgun project has been deposited at DDBJ/ENA/GenBank underthe under the accession JBPNYO000000000. The version described in this paper is version JBPNYO010000000. The NCBI BioProject accession number is PRJNA1284547. The read data (same BioProject, PRJNA1284547) are available in the SRA under the accession number SRR34355927.
